# Expression of Transient Receptor Potential Ankyrin 1 and Transient Receptor Potential Vanilloid 1 in the Gut of the Peri-Weaning Pig Is Strongly Dependent on Age and Intestinal Site

**DOI:** 10.3390/ani10122417

**Published:** 2020-12-17

**Authors:** Elout Van Liefferinge, Noémie Van Noten, Jeroen Degroote, Gunther Vrolix, Mario Van Poucke, Luc Peelman, Chris Van Ginneken, Eugeni Roura, Joris Michiels

**Affiliations:** 1 Laboratory for Animal Nutrition and Animal Product Quality (LANUPRO), Department of Animal Sciences and Aquatic Ecology, Ghent University, 9000 Ghent, Belgium; noemievannoten@gmail.com (N.V.N.); jerdgroo.degroote@ugent.be (J.D.); joris.michiels@ugent.be (J.M.); 2 Department of Veterinary Medicine, Faculty of Pharmaceutical, Biomedical and Veterinary Sciences, University of Antwerp, 2000 Antwerp, Belgium; Gunther.Vrolix@uantwerpen.be (G.V.); chris.vanginneken@uantwerpen.be (C.V.G.); 3 Laboratory of Animal Genetics, Faculty of Veterinary Medicine, Ghent University, 9000 Ghent, Belgium; mario.vanpoucke@ugent.be (M.V.P.); Luc.Peelman@ugent.be (L.P.); 4 Centre for Nutrition and Food Sciences, Queensland Alliance for Agriculture and Food Innovation, The University of Queensland, Saint Lucia 4072, Australia; e.roura@uq.edu.au

**Keywords:** TRPA1, TRPV1, weaning, pig, gastrointestinal tract, gut hormones

## Abstract

**Simple Summary:**

Weaning is a critical event for the piglet, contributing to aberrant gut function and resulting in reduced barrier function and retarded protein digestion. The gut is able to “sense” nutrients and release gut hormones to regulate digestive processes. To that end, various gastrointestinal cell types possess transient receptor potential channels that are involved in regulating gastric motility and secretion. Herbal compounds, currently used in pig nutrition as antibiotic alternatives, are able to activate these channels and could potentially aid digestion. However, these channels have not been characterized in the gut of the pig and their ability to release gut hormones has never been explored. This study’s objective was to characterize TRPA1 and TRPV1 in the pig’s gut and explore their potential to modulate gastric function. A gene expression study was performed on tissues obtained from different locations in the guts of piglets of varying age. Moreover, the ability to secrete peptide hormones was investigated by characterizing them on enteroendocrine cells. Both channels were found to be expressed in the mucosa of the porcine gut, strongly dependent on age and location. Moreover, the endocrine nature of both channels was confirmed, indicating their possible role in gut hormone release and the regulation of gastric emptying.

**Abstract:**

Transient receptor potential (TRP) channels contribute to sensory transduction in the body, agonized by a variety of stimuli, such as phytochemicals, and they are predominantly distributed in afferent neurons. Evidence indicates their expression in non-neuronal cells, demonstrating their ability to modulate gastrointestinal function. Targeting TRP channels could potentially be used to regulate gastrointestinal secretion and motility, yet their expression in the pig is unknown. This study investigated TRPA1 and TRPV1 expression in different gut locations of piglets of varying age. Colocalization with enteroendocrine cells was established by immunohistochemistry. Both channels were expressed in the gut mucosa. TRPV1 mRNA abundance increased gradually in the stomach and small intestine with age, most notably in the distal small intestine. In contrast, TRPA1 exhibited sustained expression across ages and locations, with the exception of higher expression in the pylorus at weaning. Immunohistochemistry confirmed the endocrine nature of both channels, showing the highest frequency of colocalization in enteroendocrine cells for TRPA1. Specific co-localization on GLP-1 immunoreactive cells indicated their possible role in GLP-1 release and the concomitant intestinal feedback mechanism. Our results indicate that TRPA1 and TRPV1 could play a role in gut enteroendocrine activity. Moreover, age and location in the gut significantly affected gene expression.

## 1. Introduction

It is well described that the gastrointestinal tract (GIT) can sense nutrients and other luminal factors through a chemosensory system and hence transmit signals to orchestrate GIT motility, nutrient absorption, the release of gut hormones and neurotransmitters involved in the regulation of digestive processes, and energy and glucose homeostasis [1,2,3]. To that end, various cell types such as afferent neurons, enteroendocrine cells, and enterocytes present in the GIT possess transporters, channels, and G-protein-coupled receptors that are activated by diverse stimuli. The transient receptor potential (TRP) family, which includes non-selective calcium permeable channels, detect and transmit signals and are predominantly distributed in, but not restricted to, afferent neurons. They contribute to sensory transduction, where present, and can be triggered by a range of environmental factors, such as extreme temperatures, pH changes, or by chemical agents [4,5,6,7,8]. The mammalian TRP family consists of 28 members divided into six subfamilies, classified as ankyrin (TRPA), canonical (TRPC), melastatin (TRPM), mucolipin (TRPML), polycystin (TRPP), and vanilloid (TRPV). They all consist of four subunits containing six transmembrane segments with a hydrophilic loop between the two last segments forming the ion-conducting pore. The amino acids located before the pore confer the channels’ selectivity [9].

Transient receptor potential ankyrin 1 (TRPA1) channel is the sole member of the mammalian TRPA subfamily and is characterized by multiple *N*-terminal ankyrin repeats [10]. This transmembrane protein is expressed by somato-sensory neurons (such as the trigeminal nerve), associated with perception of pain, cold, hot, and pungent irritants. It is invariably co-expressed with the transient receptor potential vanilloid 1 channel (TRPV1) [11]. TRPV1 is a member of the TRPV or vanilloid receptor subfamily and acknowledged as the capsaicin receptor. Activation by capsaicin on somatosensing nerve endings in the mouth induces a flow of sodium and calcium ions through the channel into the cell, depolarizing nociceptive neurons, leading to an action potential which results in the sensation of spiciness [12]. In addition, TRPV1 plays a key role in many other sensory functions, in detecting a large array of noxious stimuli, and was also found in vagal, splanchnic, and pelvic visceral afferents, implicated in GIT mechanosensory functions and visceral hypersensitivity [13]. Recent identification of TRPA1 expression in non-neuronal cells such as gastrointestinal enteroendocrine cells in rodents gives evidence of additional ways to modulate gastrointestinal function [14,15,16,17]. The gut endocrine system acts as the primary sensor of ingested food and secretes an array of gut hormones which act in concert to modulate multiple physiological responses, including gastrointestinal motility and secretion, glucose homeostasis, and appetite. TRPA1 was shown to be expressed in mouse and human duodenal mucosa [18], enterochromaffin cells [15], I-cells [18], and L-cells [16]. Furthermore, TRPV1 is expressed on gastrin, chief and parietal cells in the stomach, on epithelial cells of the small intestinal mucosa of several species [19,20], and in the murine enteric nervous system [21].

Recently, both cation channels have attracted significant attention as targets for herbal compounds, which have been widely used since ancient times to enhance the taste and flavor of foods and to stimulate digestive functions [22,23]. According to Nozawa et al. [15], treatment of enterochromaffin cells with TRPA1 agonists allyl-isothiocyanate and cinnamaldehyde resulted in the release of serotonin. Moreover, stimulation of STC-1 cells with allyl-isothiocyanate increased intracellular calcium and significantly stimulated cholecystokinin secretion [18]. The administration of methyl syringate suppressed cumulative food intake and gastric emptying in mice [24]. These authors showed that this response was TRPA1-mediated since HC-030031, a selective TRPA1 antagonist, inhibited the compound’s induced reduction of food intake and delayed gastric emptying. Moreover, plasma peptide tyrosine tyrosine (PYY) levels were upregulated. TRPV1’s specific location in the epithelium suggests involvement in hydrochloric acid production of the stomach [20,25]. Many of the herbal compounds currently used in animal feeds are used primarily for their antibacterial and antioxidant effects [26,27,28]. However, since these compounds act as modulators for TRPA1 and TRPV1, and are suggested to affect GIT function, specifically targeting these channels in the pig might be appealing.

In commercial swine production, the weaned piglet is subjected to environmental, physiological, and nutritional stresses [29]. This commonly results in reduced feed intake and altered histo-morphological characteristics, reduced brush-border enzyme activities, and transiently reduced barrier function of the small intestine (SI), ultimately leading to maldigestion and malabsorption and risk for infection [29,30,31,32]. An important observation in the weaned piglet is that protein digestibility is reduced, not in the least by the mentioned alterations in the small intestine but also by the inability to adequately acidify gastric contents, hampering proper pepsin activity [28,33]. Altogether, reduced foregut protein digestion leads to more undigested proteins reaching the hindgut in the post-weaning period. Since the metabolism of proteinaceous materials by microbiota leads to an increase in potentially toxic substances such as ammonia, amines, indoles, phenols, and branched-chain fatty acids, it is well accepted that protein fermentation contributes significantly to post-weaning diarrhea [34,35,36]. Bikker et al. showed this by feeding weaned piglets a lower level of crude protein with higher digestibility, which resulted in lower ammonia concentrations in the SI and decreased plasma urea nitrogen, ammonia nitrogen, and volatile fatty acids in the ileal digesta and a reduced diarrhea incidence [37].

As described above, targeting TRPA1 and TRPV1 could potentially lead to increased gastric secretions, reduced gastric emptying rates, and an increase in digestive enzyme activities. These effects could eventually improve protein digestion, gut health, and reduce pathogen proliferation as well as antibiotic usage in the pig industry. TRPA1 and TRPV1 have not been described in the GIT of pigs, and recent studies have revealed species-specific activation or blockade of TRPA1 by ligands. For example, Xiao et al. (2008) showed that human TRPA1 is activated by menthol, whereas TRPA1 from non-mammalian species is insensitive to this herbal compound [38,39,40,41]. Considering this, an in-depth characterization of these channels in the GIT of the pig, combining RT-qPCR [42] and immunohistochemistry (IHC), was performed. The latter technique was used to verify the endocrine nature of these channels, by investigating colocalization with enteroendocrine cells. The potential to modulate gastric function was explored by double staining specifically with GLP-1 positive cells, a humoral mediator of the “ileal brake” exerting inhibition of the upper gastrointestinal function.

## 2. Materials and Methods

### 2.1. Animals and Sampling Procedures

The study was conducted in accordance with the ethical standards and recommendations for the accommodation and care of laboratory animals, covered by the European Directive 2010/63/EU on the protection of animals used for scientific purposes and the Belgian Royal Decree KB29.05.13 on the use of animals for experimental studies. No ethical approval was required for this trial as animals were kept under farm practices without interventions causing harm equivalent to, or higher than, that caused by the introduction of a needle in accordance with good veterinary practice, and because animals were killed solely for the use of their organs or tissues (2010/63/EU). A total of 42 piglets (Topigs × Piétrain) were used from a commercial swine farm (Bissegem, Belgium). Prior to weaning, piglets consumed freely sow milk. Commercial weaner diets were fed after weaning. Piglets belonged to 6 different litters; 7 median body weight piglets from each litter were selected. Piglets were sampled before, around, and after weaning. More specific, six piglets were sampled at 7 different time points: 10 days before weaning (w) (w − 10); at weaning (w); 4 h (h) post-weaning (w + 4 h); and 2, 5, 14, and 28 days post-weaning (w + 2, w + 5, w + 14, and w + 28, respectively). These corresponded to 13, 23, 23 (4 h separated from sow), 25, 28, 37, and 51 days of age, respectively. Piglets were euthanized by induction of terminal anesthesia with intra-peritoneal sodium pentobarbital (90 mg kg^−1^ body weight), followed by exsanguination. Subsequently, the entire GIT was removed and tissue samples collected. The stomach was incised along the greater curvature and divided longitudinally into two parts. The contents were removed and each half was divided into cardiac, fundic, and pyloric regions. From the parietal half of the stomach, samples (2 cm × 6 cm) were excised from the center of each region, rinsed with saline, and placed on an ice-cold surface. By sharp dissection, the mucosa was separated from the underlying muscle. From the visceral half, three subsamples were taken at similar location, by cutting a piece from each region (2 cm × 6 cm), immersed into 4% formaldehyde for 18–24 h at room temperature for IHC. Three different regions from the SI were sampled, i.e., at 2.5%, 25%, and 75% of total SI length, further referred to as SI1, SI2, and SI3. A small piece (6 cm) from each location was taken and immersed in 4% formaldehyde for 18–24 h at room temperature for IHC. A second segment (30 cm) was collected, rinsed with saline, and placed on an ice-cold surface. The tissue was dissected along its length and mucosa was obtained by gently scraping with a glass slide. All mucosal samples were immediately snap-frozen in liquid nitrogen and stored at −80 °C.

### 2.2. RNA Isolation and Reverse-Transcription Quantitative Real-Time PCR

The procedures as described by Wang et al. [43] were followed. In brief, mucosal total RNA was isolated from snap-frozen stomach and SI samples using the Bio-Rad Aurum Total RNA Fatty and Fibrous Tissue Kit (Bio-Rad Laboratories, Inc., Herculas, CA, USA). An on-column DNase treatment was performed to remove possible genomic DNA (gDNA) contamination (10 U RQ1 DNase diluted in 70 µL DNase dilution buffer, 30 min at 37 °C). The concentration (ranging between 200 and 1200 ng/mL) and purity (optical density 260A/280A ranging between 1.95 and 2.25) were analyzed with the NanoDrop ND-1000 (NanoDrop Technologies, Thermo Scientific, Wilmington, DE, USA). The 18S and 28S bands were evaluated by loading a 0.8% agarose gel, in order to check RNA integrity. Sharp bands including a background smear were required to be visible. The absence of gDNA contamination was verified by performing a minus reverse-transcription control PCR using YWHAZ primers (Table 1). Then, 1 µg gDNA free RNA from each sample was converted to cDNA by using the ImProm-II cDNA synthesis kit (Promega, Madison, WI, USA), containing both OligodT and Random Hexamer primers. The obtained cDNA was diluted 10 times with molecular-grade water. Finally, a verification of the reverse transcription reaction was performed through a control PCR using 2 µL of the diluted cDNA and the YWHAZ primers as previously mentioned. Primers amplifying the target genes (TRPA1 and TRPV1; Table 1) were designed using NCBI’s PrimerBLAST, based on the common sequence which will amplify all described transcript variants of that specific gene family member. The secondary structures in the target sequences were analyzed using mfold [44]. Agarose gel (2%) electrophoresis was performed to show successful amplification with specific primer sets and template (Figure S1). RT-qPCR was performed on the CFX96 Touch Real-Time PCR Detection System (Bio-Rad Laboratories, Inc.). Then, 2 µL cDNA template was added to obtain a total volume of 10 µL containing 2× KAPA SYBR FAST qPCR Kit Master Mix (Kapa Biosystems, Inc., Wilmington, MA, USA) and 0.5 µM each of forward and reverse primers. The following protocol was used for RT-qPCR: (1) enzyme activation and initial denaturation (95 °C for 3 min); (2) denaturation/annealing/extension and data acquisition, repeated 40 cycles (95 °C for 20 s, 40 s at annealing temperature depending on primer); and (3) melt curve analysis from 70 to 90 °C, with 0.5 °C increment every 5 s. A fivefold dilution series (5 points) of cDNA and a no template control was included in each run to determine PCR efficiency by constructing a relative standard curve. In this study, PCR efficiencies per gene were between 97% and 102%. The Cq values (calibrated averages of the technical duplicates) were transformed into quantities, by using the delta-Cq formula ${\left(1+\frac{efficiency}{100}\right)}^{\Delta CT\left(highest-sample\right)}$, with the highest expression level set to 1. The relative expression for each target gene (TRPA1 and TRPV1; Table 1) was expressed as a ratio of the transformed Cq-value from the target gene to the geometric mean of the transformed Cq-values from the three reference genes. The selection of reference genes was done by using three commonly used reference genes in pig stomach and small intestine: HPRT1, YWHAZ, and RPL4 (Table 1) [45].

### 2.3. Immunohistochemistry

#### 2.3.1. TRPA1 and TRPV1 in Enteroendocrine Cells

After fixation, tissues were washed three times with phosphate-buffered saline (PBS) for 30 min, dehydrated, embedded in paraffin, and 4 μm sections were cut. Following deparaffinization and rehydration, antigen retrieval using a citrate buffer (pH 6.0) was performed, placing the sections for 20 min in a pressure cooker. After washing with tris-buffered saline (TBS), sections were treated for 10 min with 3% H_2_O_2_ in tris-buffered saline (TBS) at room temperature to block endogenous peroxidase activity, washed with TBS, and subsequently incubated for 30 min at room temperature with 20% normal goat serum (in TBS) in order to minimize non-specific antibody binding (30 min). Without washing, sections were incubated with the primary polyclonal antibody (Table 2) diluted in in TBS containing 0.3% Triton X-100 and 1% bovine serum albumin (BSA) overnight at 4 °C. Both TRPA1 and TRPV1 primary antibodies share high homology compared to humans by sequence blast (>80%). After washing with TBS, incubation with biotinylated polyclonal goat anti-rabbit IgG and streptavidin horseradish peroxidase (HRP) were each carried out for 30 min at room temperature. Immunoreactivity (IR) was visualized by applying 3,3′-diaminobenzidine. After washing with phosphate-buffered saline (PBS), slides were incubated for 2 h with chromogranin A (CgA) polyclonal antibody, as a general enteroendocrine cell marker, at room temperature. After rinsing, sections were incubated for 1 h with an Envision Mouse + system-HRP labeled polymer, and positive reactions were visualized by incubating for 5 min with 3-amino-9-ethylcarbazole+ (AEC+) solution at room temperature. Controls consisted of (a) sections incubated with TBS containing 0.3% Triton X-100 and 1% BSA, instead of the specific primary antibody, and (b) sections incubated with normal rabbit serum at the same dilution as the primary antibody. Negative control slides (omitting the primary antibody) were processed simultaneously in the same session to eliminate inter-experimental variations and yielded non-detectable staining, showing specificity of the staining results. As a positive control, duodenal tissue obtained from mice was stained, following the same procedures as described, and checked for similar expression patterns. The contribution of TRPV1- or TRPA1-IR cells to the total amount of endocrine cells (IR for CgA) in the same surface area, defined as the percentage of colocalization, was determined by counting at least 100 CgA-IR cells (IR for both TRPA/TRPV1 and CgA or IR for CgA only) and calculating the number of double-stained cells (IR for both CgA and TRPV1/TRPA1). Following this, the number of IR cells for TRPA1 and TRPV1 that were not colocalized with CgA-IR cells were counted and expressed relative to the total number of TRPV1- or TRPA1-IR cells (IR for both TRPA1/TRPV1 and CgA and IR for TRPA1/TRPV1 only). To confine the number of samples, only samples originating from 23(w) and 51(w + 28)-day-old piglets were used for the IHC study. For the quantitative analysis, an Olympus BX50 microscope connected to a computer running the software program B-cell (Olympus Belgium) was utilized.

#### 2.3.2. TRPA1 and TRPV1 Colocalization with GLP-1 Positive Cells

The same procedures as described above were followed for TRPA1 and TRPV1 double-staining with GLP-1. Instead of CgA, slides were incubated for 2 h with mouse monoclonal GLP-1 at room temperature. After rinsing, sections were incubated for 1 h with envision Mouse + system-HRP labeled polymer, and positive reactions were visualized by incubating for 5 min with 3-amino-9-ethylcarbazole+ (AEC+) solution at room temperature. Negative control slides (omitting the primary antibody) were processed simultaneously in the same session to eliminate inter-experimental variations and yielded non-detectable staining, showing specificity of the staining results. As a positive control, duodenal tissue obtained from mice was stained, following the same procedures as described, and checked for similar expression patterns.

### 2.4. Statistics

Normality of data and homogeneity of variance were tested using the Brown–Forsythe test in SAS Enterprise Guide 6 (SAS Institute, Cary, NC, USA). ANOVA using the GLM procedure of SAS with a 2-level full factorial design was carried out: age, location in GIT, and interaction, using animal as experimental unit (*n* = 6). Piglet’s litter did not exhibit a significant effect and was not further included in the model. Multiple comparisons were performed by a Tukey test. Data were expressed as means and SEM, and *p* < 0.05 was considered significant.

## 3. Results

### 3.1. TRPA1 and TRPV1 Expression in the Gut

**Figure 1:**
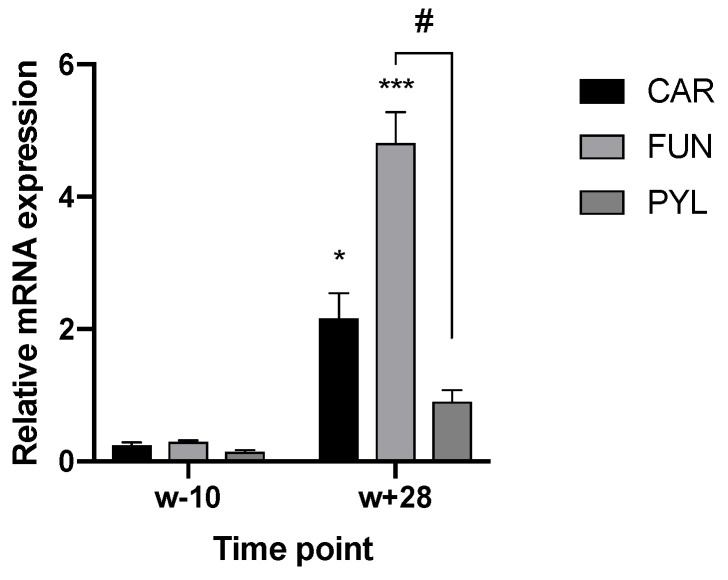
Relative TRPV1 mRNA level in the three regions of the stomach (cardia (CAR), fundus (FUN) and pylorus (PYL)), 10 days before (w − 10) and 28 days after (w + 28) weaning. Significant differences within stomach regions from other time points are indicated with asterisks: * *p* < 0.05; *** *p* < 0.001. Significant differences within time points between stomach regions are expressed with a hash: # *p* < 0.05. Number of replicates per treatment was *n* = 6. Error bars express the SEM.

**Figure 2:**
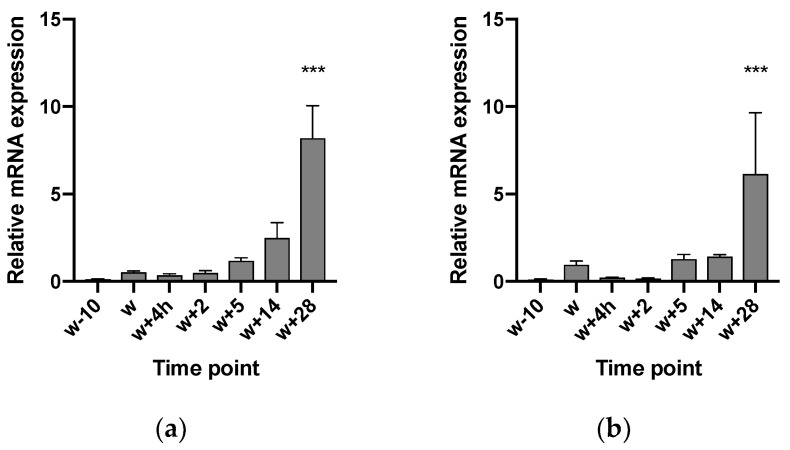
Relative TRPV1 mRNA level at (**a**) 25% and (**b**) 75% of total small intestinal (SI) length, 10 days before weaning (w − 10); at weaning (w); 4 h (h) post-weaning (w + 4 h); and 2, 5, 14, and 28 days post-weaning (w + 2, w + 5, w + 14, and w + 28, respectively). Significant differences from other time points are indicated with asterisks: *** *p* < 0.001. Number of replicates per treatment was *n* = 6. Error bars express the SEM.

**Figure 3:**
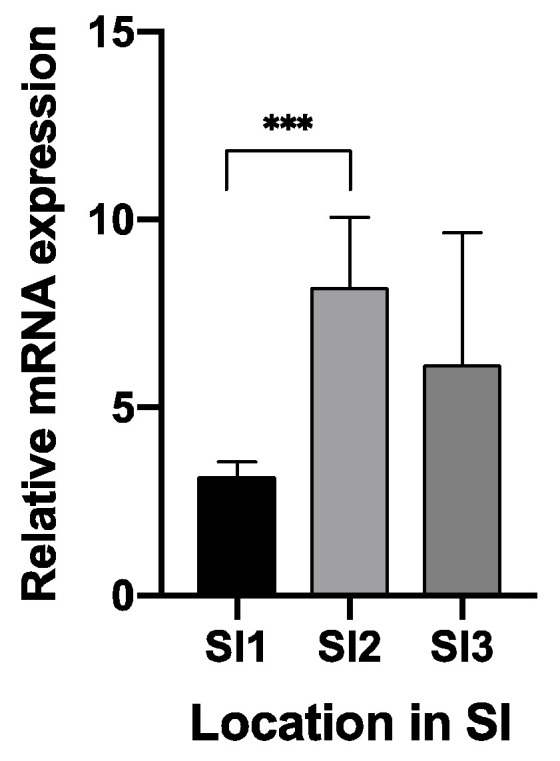
Relative TRPV1 mRNA level 28 days post weaning (w + 28) in the three regions of the SI (SI1: 2.5% of total SI length; SI2: 25% of total SI length; SI3: 75% of total SI length). Significant differences between regions are indicated with asterisks: *** *p* < 0.001. Number of replicates per treatment was *n* = 6. Error bars express the SEM.

**Figure 4:**
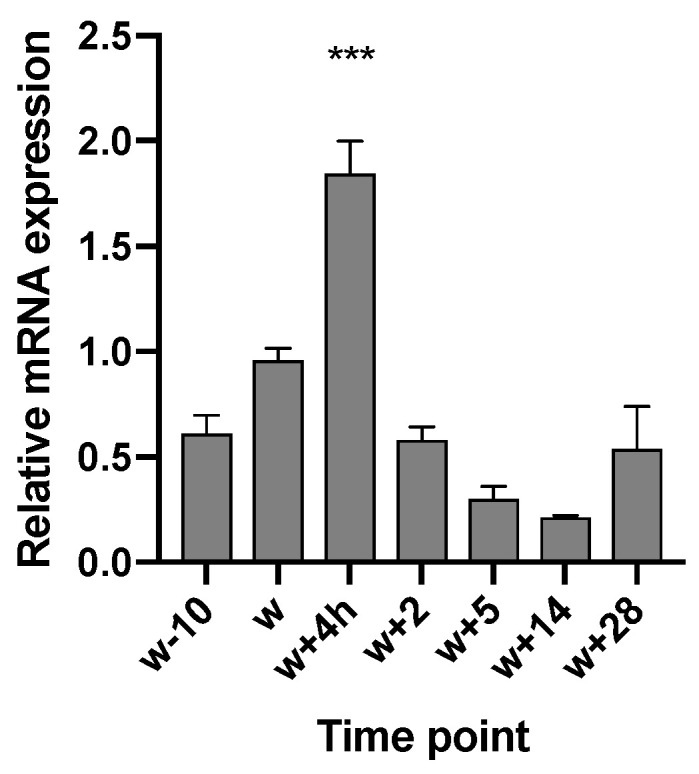
Relative TRPA1 mRNA level in the pyloric region (PYL), 10 days before weaning (w − 10); at weaning (w); 4 h (h) post-weaning (w + 4 h); and 2, 5, 14, and 28 days post-weaning (w + 2, w + 5, w + 14, and w + 28, respectively). Significant differences from other time points are indicated with asterisks: *** *p* < 0.001. Number of replicates per treatment was *n* = 6. Error bars express the SEM.

Using RT-qPCR, the expression of TRPA1 and TRPV1 in the GIT has been confirmed and described. Regarding TRPV1, age (*p* < 0.001), GIT location (*p* = 0.006), and the interaction between both parameters (age × location) (*p* < 0.001) had an effect on the mRNA level. In the stomach, expression levels varied between compartments and age. Piglets at 28 days post-weaning (w + 28) showed higher expression both in cardia (CAR) and fundus (FUN), compared to the expression level 10 days before weaning (w − 10). At 28 days post-weaning, the highest expression was found in the fundic region (Figure 1). In the SI, the expression level increased gradually in all three regions, although the increase was most notable in SI2 (Figure 2a) and SI3 (Figure 2b). W + 28 piglets exhibited a 15- and 12-fold higher mRNA level in SI2 and SI3, respectively, as compared to newly weaned counterparts (w + 4 h) (Figure 2a,b). At 28 days post-weaning (w + 28), the highest expression was found in SI2 (Figure 3). Regarding TRPA1, age (*p* < 0.001), location in GIT (*p* = 0.005), and the interaction (*p* < 0.001) had an effect on the expression level. TRPA1 showed sustained expression independent of age and location, except for higher expression in the pyloric region at weaning (w + 4 h). In this region, higher expression was found for piglets euthanized 4 h after weaning compared to piglets sampled at weaning age, but without 4 h separation from the sow (w) (Figure 4).

### 3.2. Immunohistochemistry

#### 3.2.1. TRPA1 and TRPV1 on Enteroendocrine Cells

**Figure 5:**
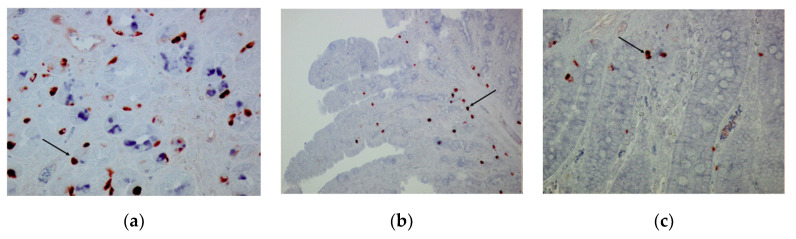
TRPV1 (blue) and CgA (red)-IR cells in the gastrointestinal tract (GIT). Cell indicated with a black arrow shows immunoreactivity for both antibodies. (**a**) Fundus, weaning (w), ×400; (**b**) SI2 (25% of total SI length), weaning (w), ×200; (**c**) SI3 (75% of total SI length), 28 days post-weaning (w + 28), ×400.

**Figure 6:**
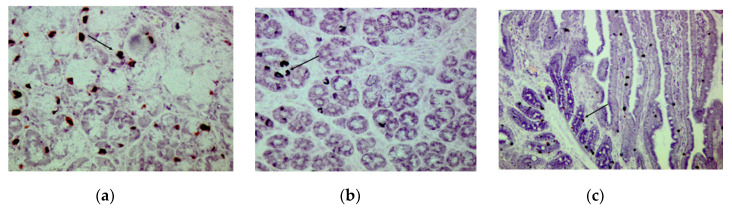
TRPA1 (blue) and CgA (red)-IR cells in the GIT. Cell indicated with a black arrow shows immunoreactivity for both antibodies. (**a**) Pylorus, weaning (w), ×400; (**b**) pylorus, 28 days post-weaning (w + 28), ×400; (**c**) SI2 (75% of total SI length), weaning (w), ×200.

**Figure 7:**
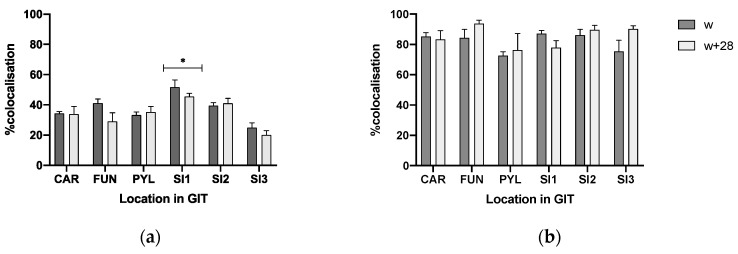
%CgA-IR cells IR for (**a**) TRPV1 and (**b**) TRPA1 at different locations in the GIT (CAR: cardia, FUN: fundus, PYL: pylorus, SI1; SI2; SI3: resp. 2.5, 25, 75% of total SI length) at weaning (w) and 28 days post-weaning (w + 28). Significant differences between regions are indicated with asterisks: * *p* < 0.05, pooling the two time points. Number of replicates per treatment was *n* = 6. Error bars express the SEM.

**Figure 8:**
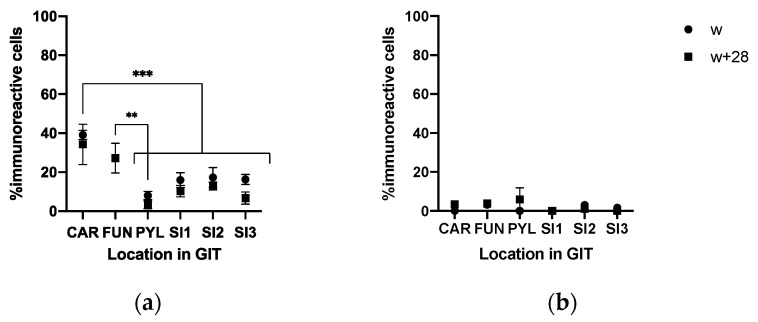
(**a**) %TRPV1-IR cells and (**b**) %TRPA1-IR cells that did not colocalize on CgA-IR cells (CAR: cardia, FUN: fundus, PYL: pylorus, SI1; SI2; SI3: resp. 2.5%, 25%, 75% of total SI length) at weaning (w) and 28 days post-weaning (w + 28). Significant differences between regions are indicated with asterisks: ** *p* < 0.01; *** *p* < 0.001, pooling the two time points. Number of replicates per treatment was *n* = 6. Error bars express the SEM.

CgA-immunoreactive (IR) cells were detected in the three glandular regions of the gastric mucosa (Figure 5a and Figure 6a,b) and along the SI (Figure 5b,c and Figure 6c) at w and w + 28. Both in stomach and SI, CgA-IR cells were observed that also stained positive for TRPV1 and TRPA1. Age (w versus w + 28) did not have an effect on the percentage of colocalization between CgA and TRPV1 and TRPA1 or on the percentage of TRPA1- and TRPV1-IR cells that did not stain in CgA-IR cells (Figure 7 and Figure 8). In both cases, only location in the GIT had an effect on the percentage of colocalization (*p* < 0.001) and on the percentage of single TRPA1- and TRPV1-IR cells (*p* < 0.001). Regarding TRPV1, the percentage of colocalization remained constant in the three glandular regions of the stomach and was highest in SI1 (48.6 ± 2.9%) (Figure 7a). The percentage TRPV1-IR cells that did not colocalize in CgA-IR cells (Figure 8a) was highest in the cardia (43.26 ± 3.79%) and fundus (27.17 ± 3.96%) and lowest in the pylorus (5.60 ± 4.04%). TRPA1-IR cells showed overall high (>72.6%) colocalization with CgA-IR cells, in all GIT locations, without any significant differences (Figure 7b). The number of TRPA1-IR cells that did not colocalize with CgA-IR cells remained low (<3.17%) in all regions of the stomach and SI (Figure 8b). A higher density of cells IR for both TRPA1 or TRPV1 with CgA was observed in the crypts of the SI compared to the epithelium of the villi (Figure 5b and Figure 6c).

#### 3.2.2. TRPA1 and TRPV1 on GLP-1 Immunoreactive Cells

**Figure 9:**
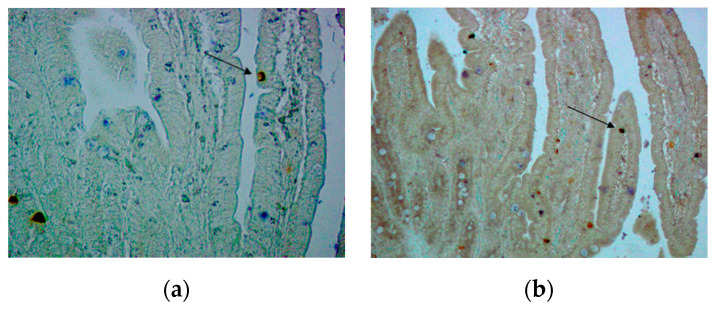
(**a**) TRPA1-(blue), and (**b**) TRPV1-(blue) double-stained with GLP-1(red)-IR cells in SI3 (75% of total SI length) at weaning (w). Cell indicated with a black arrow shows immunoreactivity for both antibodies. ×400 (**a**), ×200 (**b**).

GLP-1 positive cells were found in all three small intestinal locations. The highest density was observed in the distal SI (SI3). Similarly, GLP-1-IR cells in the three SI locations were found to express TRPA1 and TRPV1 (Figure 9).

## 4. Discussion

This is the first report describing the differential gene expression and TRP channel distribution in the GIT of young pigs. Using RT-qPCR, it was observed that TRPA1 and TRPV1 were expressed in stomach and SI. Previous studies combined RT-qPCR results with in situ hybridization, both based on the detection of mRNA [14,15,46]. Since measurements taken from mRNA and protein levels are complementary, combining RT-qPCR with IHC to characterize TRPV1 and TRPA1, specifically in the pig as target species, supplies additional information related to the translation from mRNA to proteins [47]. This was confirmed by immunological staining showing that TRPA1 and TRPV1 proteins were present in epithelial cells of the stomach and SI. This distribution suggests an important role for these TRP channels in the GIT of the pig.

### 4.1. TRPV1 Expression Is Age and Location-Dependent and Colocalizes with GLP-1-IR Cells

The highest age-related increase in TRPV1 mRNA abundance was detected in the fundus and distal SI. Immunohistochemistry indicated the colocalization with CgA-IR cells and, more specifically, with GLP-1-IR cells, which are most abundant in the distal SI [48]. Furthermore, Van Ginneken et al. [48] showed an increase in GLP-1-IR cells in the jejunum and ileum of young pigs, according to the age, which is confirmed in this study. This suggests that TRPV1 is constitutive to this type of cell and reveals their enteroendocrine nature. The highest regulatory role for TRPV1 is thus expected to occur in the distal SI, probably in the release of gut peptides such as GLP-1, which mediates the “ileal brake” mechanism and stimulates glucose-dependent pancreatic insulin secretion. Dietary factors such as glucose, fatty acids, and fiber are known to increase the expression and to stimulate the release of GLP-1 in rodents [49,50,51]. The increase in feed intake with age observed here could thus be a possible explanation for the gradual upregulation of both GLP-1 and TRPV1. Piglets are naturally weaned at a much older age than in commercial settings. In nature, weaning involves a less abrupt switch from a milk-based to a solid diet. Suckling piglets are not yet exposed to TRPV1 channel activators, such as capsaicin, that possibly could be harmful. Once piglets become older, the necessity of having these channels—to detect harmful substances—increases, as the animals become more exposed to these compounds. TRPV1 has previously been described in parietal cells in the human stomach [20]. The high number of TRPV1 immunoreactive cells that did not stain with CgA cells in the cardia and fundus, and the age-dependent increase in the expression of the genes of interest in these two regions, suggests their location on parietal and chief cells, which could indicate a possible role in HCl production and pepsinogen release. Faussone-Pellegrini et al. indicated that the mitochondrial location in human parietal cells might be related to the specific function of this cell type, i.e., the HCl secretion, by considering that: (1) TRPV1 is a non-selective cation channel involved either in Ca^2+^ binding or mobilization, (2) Ca^2+^ is stored in the mitochondria of the parietal cells and is necessary to produce HCl, (3) mitochondria are involved in cation production, and (4) TRPV1 is activated by low pH as well as by capsaicin [20,52,53]. Therefore, mitochondrial TRPV1 (as suggested by the cytoplasmatic staining) could be one of the steps in an intracellular circuit of autoregulation of HCl production by the parietal cells, yet this needs further confirmation. In the pyloric part, the low percentage of TRPV1-IR cells that did not colocalize with CgA indicates that they are mostly of endocrine nature. Since gastrin (G−) cells are abundant in this part of the stomach, the occurrence of TRPV1 on these cell types may indicate the channel’s role in gastrin release [54]. This peptide hormone stimulates the release of histamines by enterochromaffin-like cells, which consequently acts in a paracrine manner on parietal cells, stimulating them to secrete H+ ions. All previous findings suggest an important role of TRPV1 in the gastric HCl production and pepsinogen release in the pig.

### 4.2. TRPA1 Exhibits Peak Expression in Pylorus around Weaning and Highly Colocalizes with Enteroendocrine Cells

TRPA1 shows an age-independent sustained expression pattern. However, at weaning, there was a significant increase in the pyloric region. In addition, in this gastric region, a high colocalization with CgA-IR cells was observed, including a low number of TRPA1-IR cells lacking the endocrine nature. G-cells (gastrin-releasing cells) are the most dominant endocrine subtype in the pyloric region [54]. The act of removing the piglets from the sow caused an upregulation in TRPA1 expression. This was emphasised by the observation that piglets sampled at the same age, without prior separation from the sow, did not show this elevated TRPA1 mRNA abundance in the pylorus. Our data are consistent with the results published by Camacho et al., also observing higher TRPA1 expression in the pyloric compared to the cardiac region in mice [14]. Furthermore, they observed that the expression pattern of TRPA1 is similar and proportional to ghrelin expression being higher in the pyloric than cardiac epithelia. This, combined with the high in vitro expression of TRPA1 in ghrelin secreting cells (MGN 3-1) with the decrease in ghrelin secretion partially through TRPA1 activation, allowed them to explain the involvement of TRPA1 in gastric emptying and food intake. Moreover, the high number of TRPA1-positive cells in the pylorus is consistent with the role of the distal stomach in sensing nutrients [55]. Asakawa et al. [56] showed that ghrelin expression in the stomach was increased by tail pinch stress and starvation stress in mice. This suggests that this gut peptide may have a role in mediating both neuroendocrine and behavioural responses to stressors and that the stomach could play an important role in the regulation of appetite and anxiety. Notably, the range of newly found mediators of TRP channels includes oxidative stress. Endogenous reactive oxygen species, generated during mitochondrial oxidative metabolism as well as in cellular response to cytokines and bacterial invasion, can activate TRP channels [57]. TRPA1 has a higher sensitivity to these compounds than TRPV1 due to its higher number of cysteine residues (prone to oxidation) in the longer outstretched arm of the *N*-terminus [58]. As shown by our data, TRPA1 expression is affected by the act of weaning the piglet, where the immature animal is subjected to a variety of stressors linked to changes in social structure, diet, and environment [27]. Nevertheless, the causal link between higher mRNA and stress and anxiety is yet only proven in other species than the pig and should thus be further confirmed in the pig. TRPA1 showed a high (>75.4%) colocalization with enteroendocrine cells in the three parts of the SI, which indicates a possible mediation in hormone release. The hormone released depends on the specific enteroendocrine cell type. However, recent work implied that these cell lineages in the SI exhibit a more flexible hormone repertoire than previously proposed [59]. Furthermore, higher colocalization of both channels with enteroendocrine cell types in the crypts compared to the villi was observed, which is in line with previous findings [14]. This also indicates their possible role in GLP-1 release, since Beumer et al. [59] suggested that enteroendocrine cells could switch hormone expression along the crypt-to-villus gradient. Cells expressing GLP-1 are restricted to crypts, whereas PYY enteroendocrine cells are more abundant in the villi [59].

## 5. Conclusions

Our study showed that both TRPV1 and TRPA1 channels are expressed along the GIT of pigs and supports the hypothesis that the function of both may be related to an endocrine response, possibly resulting in the release of gut peptide hormones such as GLP-1. Moreover, age significantly affected their gene and protein expression with variable expression patterns, which suggests their involvement in distinct physiological functions, such as the ability to modulate the gastric function.

This study provides substantially more knowledge and data for pigs considering TRPA1 and TRPV1 expression in the GIT. We do believe that this is a first step in exploring the possibilities of targeting TRP channels in the pig’s gut and will contribute to further research. Given that the functional implications of our findings are deduced by comparison with existing data in other species, further investigations are needed to elucidate the physiological implications of these findings in the pig and to identify which compounds are directly involved in their modulation.

## Figures and Tables

**Table 1:** Primers used for real-time PCR, annealing temperature (Ta), and amplicon length.

Gene Symbol	Accession Number	Nucleotide Sequence of Primers, 5′-3′	Ta (°C)	Product Length (bp)
HPRT1	DQ178126	Forward: CCGAGGATTTGGAAAAGGT Reverse: CTATTTCTGTTCAGTGCTTTGATGT	60	181
YWHAZ	DQ178130	Forward: ATGCAACCAACACATCCTATC Reverse: GCATTATTAGCGTGCTGTCTT	60	178
RPL4	XM_005659862	Forward: CAAGAGTAACTACAACCTTC Reverse: GAACTCTACGATGAATCTTC	58	122
TRPA1	XM_021089237.1	Forward: GAATTTACTCATTGGTTTGGCAGTTGGTG Reverse: CGGTGATGGATTTCTGATCGACCTTG	58	155
TRPV1	XM_013981216.2	Forward: GGACAGCGAGTTCAAAGACC Reverse: CCGTTTTCCACCAGAAGTGT	63	240

**Table 2:** Primary and secondary antibodies, species raised in, clonality, dilution, and research resource identifier.

	Target	Species Raised in; Clonality	Dilution	Research Resource Identifier
Primary antibodies	TRPA1	Rabbit, polyclonal	1:100	Cat#NB110-40763, Novus Bio
	TRPV1	Rabbit, polyclonal	1:200	Cat#orb13755, Biorbyt
	Chromogranin A	Mouse, polyclonal	1:200	Cat#M0869, Dako
	GLP-1	Mouse, monocloal	1:5000	Cat#A6104-1, Immun Diagnostik
Secondary antibodies	Anti Rabbit IgG	Goat, polyclonal	1:200	Cat#31823, Invitrogen
	Envision + System-HRP labeled polymer	Mouse	1:1	Cat#K4001, Dako

## Supplementary Materials

The following are available online at https://www.mdpi.com/2076-2615/10/12/2417/s1, Figure S1: Agarose gel (2%) electrophoresis shows successful amplification with specific primer sets and template.
