# Supplementary material for: Expression of Transient Receptor Potential Ankyrin 1 and Transient Receptor Potential Vanilloid 1 in the Gut of the Peri-Weaning Pig Is Strongly Dependent on Age and Intestinal Site
Source: Animals (Basel). 2020 Dec 17;10(12):2417. doi: 10.3390/ani10122417 (PMC7766004; doi:10.3390/ani10122417)
Supplement: Supplementary file 1 [file animals-10-02417-s001.pdf]

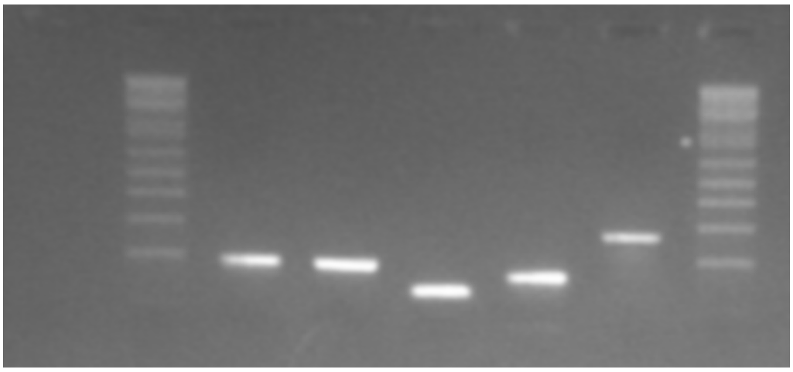

Supplementary figure 1. Agarose gel (2%) electrophoresis shows successful amplification with specific primer sets and template. (Lane 2) HPRT1 181bp, (Lane 3) YWHAZ 178bp, (Lane 4) RPL4 122bp, (Lane 5) TRPA1 155bp, (Lane 6) TRPV1 240 bp.
